# Effects of Extrusion on Energy Contents and Amino Acid Digestibility of Corn DDGS and Full-Fat Rice Bran in Growing Pigs

**DOI:** 10.3390/ani12050579

**Published:** 2022-02-25

**Authors:** Zeyu Zhang, Ge Zhang, Shuai Zhang, Jinbiao Zhao

**Affiliations:** State Key laboratory of Animal Nutrition, College of Animal Science and Technology, China Agricultural University, Beijing 100193, China; mafic2017zeyu@163.com (Z.Z.); zhangge0557@163.com (G.Z.); zhangshuai16@cau.edu.cn (S.Z.)

**Keywords:** extrusion, growing pig, energy, digestibility, amino acids

## Abstract

**Simple Summary:**

Our study showed that extrusion improves nutritive value, apparent total tract digestibility of nutrients and ileal digestibility of most amino acids in full-fat rice bran and corn distillers dried grain with solubles, which suggests that extrusion could be an effective strategy to improve nutritive values of feed by-products.

**Abstract:**

The objectives of this study were to determine the effects of extrusion on available energy, apparent total tract digestibility (ATTD) of nutrients and energy, and amino acid (AA) digestibility of full-fat rice bran (FFRB) and corn distillers dried grain with solubles (DDGS) fed to growing pigs. Methods: In Exp. 1, a total of 30 growing pigs with initial body weight (BW) of 36.0 ± 1.8 kg were fed five different diets, including one corn basal diet and four experimental diets which were formulated by 29.06% FFRB or DDGS with or without extrusion processing. In Exp. 2, 30 ileal-cannulated pigs (initial BW: 20.3 ± 1.8 kg) were fed five different diets including 40% FFRB or DDGS with or without extrusion, and a N-free diet. Results: The results showed that there were no significant differences in DE and ME contents or ATTD of GE, DM, and OM between DDGS and FFRB (*p* > 0.05), but the ATTD of CP, NDF, and ADF showed significant differences between DDGS and FFRB (*p* < 0.05). In addition, the DE and ME values (*p* < 0.01) and ATTD of GE, DM, OM, and NDF improved when pigs were fed extruded diets (*p* < 0.05), and a trend to increase the ATTD of CP and ADF was observed when pigs were fed extruded diets (*p* = 0.06 and 0.07, respectively). The AID and SID levels of CP were not different when pigs were fed diets with or without extrusion. The AID of total indispensable AA increased when pigs were fed extrusion diets (*p* < 0.05). Conclusion: Feed processing of extrusion could improve nutritive values of FFRB and DDGS.

## 1. Introduction

Extrusion has been widely used in feed processing for over 70 years [1]. It can change the physio-chemical properties of feed ingredients by applying constant moisture, pressure, and high temperature with the combination of shear force. Thus, extruded ingredients have greater nutrient utilization and reduced anti-nutritional factors [2].

Full-fat rice bran (FFRB), an important by-product of the rice milling industry, is commonly used as an alternative to energy feed material in animal diets because it contains 15–22% oil. However, the presence of anti-nutritional factors (ANFs) such as phytic acid hinders its commercial promotion and widespread application [3,4]. Corn distillers dried grains with solubles (DDGS) is a by-product of the fuel ethanol industry that can provide energy and protein for animals. However, the effects of extrusion on nutritive values of FFRB and DDGS in growing pigs is still poorly characterized. DDGS and FFRB are increasingly used to replace SBM in pig diets. There are many processing technologies to produce DDGS and FFRB which contain varying chemical components. Therefore, it is important to evaluate energy contents and amino acid digestibility of DDGS and FFRB produced in China. 

Extrusion increased the ATTD of starch and energy of faba bean diets and decreased the feed wastage, possibly due to the starch gelatinization after extrusion [5,6,7,8]. In addition, pigs fed extruded peas showed greater digestibility of starch and amino acids (AA) [9] despite the existence of various anti-nutritional factors (ANFs) in peas [10]. Extrusion decreased the total glucosinolate content and fiber fractions (NDF, ADF) in canola meal with screw speeds of 350 rpm and 450 rpm, respectively. Increasing the screw speed of the extruder to 350 rpm seems to reduce the total content of glucosinolates in canola meal (CM), and by increasing the screw speed to 450 rpm, the NDF and ADF contents decreased in CM [11]. Moreover, extrusion could improve the storability of feedstuffs [12]. When using extruded FFRB in broiler diets, significantly increased fat digestibility was observed compared with non-extruded FFRB [13]. 

Therefore, the objective of this study was to determine energy contents, the ATTD of energy and other nutrients, and the standardized ileal digestibility (SID) of amino acid (AA) in pigs fed either corn DDGS or FFRB with or without extrusion.

## 2. Materials and Methods

The FFRB and DDGS were extruded at 120–130 °C by using a high-capacity double-helix extruder under the condition of 4000 mm length and 200 mm transport screws (Yang Gong Extruder, Model TPE62S, Beijing, China). The extruder barrel temperature and the screw speed were controlled by a computer connected to the extruder to adjust the passage time to 10 s. 

### 2.1. Experimental Diets and Design

#### 2.1.1. Exp. 1

Growing pigs were fed to determine the apparent total tract digestibility (ATTD) of nutrients and energy, digestible energy (DE), and metabolizable energy (ME) of extruded or non-extruded FFRB and DDGS. A completely randomized experimental design was adopted, and 30 crossbred pigs with initial body weight of 36.0 ± 1.9 kg were used and divided into 5 treatments with 6 replicates per treatment. The duration of Exp. 1 was 12 days, including 7 days of diet adaptation and 5 days of sample collection. A total of five experiment diets were formulated and fed to the pigs. In each collection period, samples of feces and urine from each pig were collected. Formulations of the diets and chemical analysis are shown in Table 1 and Table 2. According to NRC (2012) [8], trace mineral premix was added to all meals to meet nutritional needs. According to the data of Adeola (2001) [14], the daily feeding amount of each pig was equal to 4% of its body weight and is divided into two groups to provide the same amount of feed at 08:30 and 15:30 h. All pigs had free access to water.

Pigs were maintained individually in stainless-steel metabolism boxes (1.4 m × 0.7 m × 0.6 m) at room temperature ranging from 18 to 22 °C. In order to maintain environmental hygiene, the boxes were cleaned and rinsed twice daily. Finally, animal experiments were conducted, including 7 days of dietary adaptation and 5 days of feces and urine collection. Feces were collected in plastic bags and stored in −20 °C. While collecting feces, the total amount of urine was collected in a plastic bucket connected to funnels under the metabolic cages. We added 20 mL of 10% (*V*/*V*) hydrochloric acid to the plastic bucket to fix nitrogen contained in the urine. Urine volume was recorded every day, and 10% of sub samples were collected every day and stored at −20 °C. At the end of the experiment, feces were thawed, weighted, and mixed, and subsamples were taken and dried in a forced ventilation oven at 65 °C for 72 h.

#### 2.1.2. Exp. 2

Growing pigs were fed to determine the AID and SID of crude protein (CP) and AA of extruded or non-extruded FFRB and DDGS. The diets were formulated with 40% FFRB or DDGS with or without extrusion as the sole source of nitrogen (Table 3). The N-free diet was used to determine basal ileal endogenous N losses [15]. The Cr_2_O_3_ (0.3%) was added to all diets as an indigestible marker.

In ten crossbred barrows (Duroc × Landrace × Yorkshire), the initial BW 20.3 ± 1.8 kg operation was equipped with T-cannula at the end of the ileum, which was placed separately in a metabolism box (1.4 m × 0.7 m × 0.6 m) located in the temperature-controlled room from 18 to 22 °C, assigned to a Yuden square. Phase 3 and 5 dietary treatments were designed. The three stages were 7 days each; the pre-test period was 5 days, and the ileal digesta collection period was 2 days. Ileal digesta was collected continuously from 08:00 to 17:00 h. The collection procedure here is described by Stein et al. [15]. Simply put, on days 6 and 7, a 200 mL plastic bag was fixed to the open sleeve with a tie. Whenever the digesta was filled, or at least every 30 min, the bag was removed and stored at −20 °C until analysis was required. After the experiment, the digesta samples were thawed, mixed with pigs and stage pigs, and then sampled and freeze-dried in a vacuum freeze dryer.

### 2.2. Calculations and Statistics Analysis

The DE and ME values and the ATTD of GE, DM, OM, CP, NDF, and ADF in diets were calculated using the direct method (Equation [16]; Dong et al., 2020). The AA digestibility of DDGS and FFRB samples was calculated as described by Stein et al. [15]. 

The normality of residuals and equal variances of raw data were checked using the UNIVERIATE procedure of SAS 9.2 (SAS Inst. Inc., Carry, NC, USA) to identify outliers, and no outliers were observed in the study. All data were analyzed as a 2 × 2 factorial experimental design using the GLIMMIX procedure. Each individual pig was analyzed as an experimental unit. The analyzed model included the fixed main effects of ingredient type and extrusion, and periods were included as a random effect. The LSMEANS statement was used to calculate the least squares mean for each treatment. Significant differences were considered significant if *p* < 0.05.

## 3. Results

### 3.1. Exp. 1: Energy Content and Nutrients Digestibility 

The available energy and ATTD of nutrients and energy of DDGS and FFRB with or without extrusion are presented in Table 4. No interactive effects between ingredient type (DDGS or FFRB) and processing (extrusion or non-extrusion) were observed on DE and ME contents or the ATTD of GE, DM, OM, and ADF. There were no differences in DE and ME between FFRB and DDGS. The DE and ME contents and ATTD of GE, DM, and OM in extruded diets were greater (*p* < 0.05) compared with the non-extruded diets fed to pigs. Extrusion did not affect the ATTD of NDF and ADF, but the DDGS diet showed greater ATTD of NDF and ADF compared with the FFRB diet (*p* < 0.01), and the extrusion diet tended to increase the ATTD of CP and ADF (*p* = 0.06 and 0.07, respectively).

### 3.2. Exp. 2: Cannulated Pig Trial 

There were interactive effects (*p* < 0.05; Table 5 and Table 6) on AID of His, Leu, Lys, Met, Phe, Thr, Val, Ala, Asp, Cys, Clu, Ser, and Try, and SID of Leu, Lys, Phe, Val, Asp, and Try in FFBR and DDGS diets between extrusion processing and ingredient type. Extrusion diets increased the AID of most AA, except for Arg, Trp, and Gly, compared with no-extrusion diets. Extrusion did not affect AID and SID of CP, but increased (*p* < 0.05) SID of Leu, Met, Phe, Val, Ala, Asp, Clu, Ser, and Tyr. The FFRB diet had greater AID of Arg and Glu and SID of His, but lower AID of Ile and SID of Met compared with the DDGS diet.

## 4. Discussion

Body weight was randomly selected. In addition, many publications have reported that evaluated precision of energy values and amino acid digestibility would improve as inclusion levels of tested ingredients increased; however, pig performance would be suppressed if dietary inclusion levels of FFRB and DDGS are too high because FFRB and DDGS contain high levels of fiber components. In addition, all diets were semi-pure diets in Exp. 2; dietary fiber and protein contents were provided by FFRB and DDGS. Therefore, the inclusion level of 40% FFRB and DDGS in Exp. 2, not 29.06%, was used in order to ensure reasonable nutrient levels for pigs. Of course, all results in this study are shown under conditions of specific pig body weight and inclusion levels of tested ingredients.

FFRB and DDGS are important by-products of the rice and corn industry, and have the potential to be used as alternatives to energy feed ingredients in animal diets [3,4]. Different digestibility of energy and nutrients may be caused by different chemical compositions and processing technologies [17,18]. In the extrusion process, the utilization rate of nutrients is improved through high temperature and pressure, which leads to the rupture and expansion of intact cells in the material in response to the sudden drop in pressure and temperature when leaving the extruder [19]. Extrusion has no effects on the content of nutrients and GE of both FFRB and DDGS, as previously reported by Huang (2021) [20]. The chemical compositions of DDGS and FFRB herein were consistent with previous literature results [20,21]. 

Extrusion increased starch digestibility and energy utilization by the pigs, which is one of the reasons for the increased ME [9]. Ether extract was the best predictor and positively correlated with the DE and ME. Higher EE content in FFRB resulted in higher DE content [22]. Similarly, extrusion significantly increased the DE and ME of DDGS and FFRB in our study. In pig feed formula, energy content is the largest cost [23]. In the present study, extrusion increased energy digestibility, and the DE content increased from 13.84 to 15.86 MJ/kg and ME from 13.36 to 15.24 MJ/kg. This observation was consistent with the published data that extrusion leads to the increase in ATTD of GE in field peas [24]. The increased ATTD of GE observed for extrusion diets was a result of increased AID of AA and starch [25]. The increased nutrient digestibility is probably caused by the cleavage of non-starch polysaccharides into smaller fragments, thereby substantially reducing their anti-nutritive effects [26]. Extrusion could also improve the digestibility of specific nutrients in DDGS, such as protein, by changing the structure, function, or chemical properties of DDGS [27]. The improvement of fiber digestibility may be that extrusion contributes to the redistribution of insoluble and soluble fiber components. In our study, extrusion improved DE and ME content and ATTD of GE, DM, and OM; moreover, extrusion tended to increase ATTD of CP and fiber fractions.

In previous studies, the results for the effects of extrusion on CP digestibility were not consistent in swine nutrition. Extrusion did not affect the AID of CP in FFRB fed to pigs [28]. Similarly, extrusion did not affect the ATTD and AID of CP and indispensable AA in corn fed to 20 kg pigs [29]. In our study, the extruded diet increased the AID and SID of most AA compared with the non-extruded diet, but it had no significant effects on AID or SID of CP, Lys, Trp, and Cys. The increase in dietary EE content delayed gastric emptying [30], and slower gastric emptying may lead to slower evacuation passage, thus providing a longer time for peptide and AA digestion and absorption [31]. Therefore, these results showed that extrusion is more suitable for FFRB processing than DDGS in pig diets. The suggested explanation is that FFRB has more fat and therefore a longer passage time, which made possible a better digestibility of amino acids. In this experiment, the AID levels of the essential AA of FFRB were slightly lower than those reported by Huang [27]. This difference may be due to different sources of FFRB or different extrusion conditions. At present, there are few studies focusing on the effects of extrusion on FFRB, which has less AID of AA compared with defatted rice bran [20]. This phenomenon has been improved after extrusion. The AID levels of CP in DDGS and extruded DDGS were approximately 55% and 60%, similar to previous reports [27].

The addition of oil in the diet of growing pigs not only increased the AID of AA, but also increased SID [2]. Extrusion technology can improve the effect of high fiber by-product diets on the growth performance of pigs [9]. Perhaps this result was caused by greater SID of Lys and most of AA in the FFRB after extrusion in the present study. It is reported that under low moisture content, Lys may be lost due to high temperature extrusion or shear stress [32]. Extrusion significantly increased the AID of nutritional components in DDGS diets of corn and wheat [17,33] in pigs, especially SID of AA. Denaturing of proteins by heat temperature may explain greater AID and SID of AA in the present study. Depending on the AA source, heat denatures proteins at a temperature range of 25–100 °C [34]. In addition, extrusion treatment will break the fat wall of raw materials and degrade the fat to form glycerol and free fatty acids, and free fatty acids will combine with starch and protein to form a complex to reduce the digestibility of nutrients. As the extrusion temperature rises, the synthesis of this complex will increase. When the temperature of the inner wall of the extrusion chamber exceeds 100 °C, the number of composites decreases significantly [35]. Studies have shown that the improvement of nutrient mass digestibility of raw materials by single screw extrusion is better than that by twin screw extrusion [24]. The greater shear force of the single screw extrusion process can destroy the level and structure of fat [36], indicating that it is important to know the optimal expansion equipment and conditions to achieve the maximum digestibility of fat. In this experiment, extrusion led to the decrease in digestibility of some amino acids, perhaps due to the Maillard reaction between amino acids and reducing sugar, which renders some amino acids invalid [37]. In addition, more fibers combine with lysine, which hinders the opportunity of binding with protease and reduces the digestibility of amino acids [31]. Lewis found that with the increase in water content, the in vitro digestibility of CP first decreased and then increased. The main factors affecting the CP digestibility of extruded feed were water content and extrusion temperature, followed by screw speed. The CP digestibility could be significantly increased by increasing feeding speed in corn germ extrusion [38]. However, the results from the present study indicate that extrusion effectively enhances energy and nutrient digestibility, and is beneficial for improving AID and SID of AA.

In conclusion, extrusion of FFRB and DDGS improved the digestibility of GE, OM, CP, and some AA, and thereby increased the content of DE and ME. Extrusion technologies should be considered to optimize feed utilization in pigs.

## Figures and Tables

**Table 1:** Analyzed chemical compositions of the experimental ingredients (%, as-fed basis) ^1,2^.

Item %	Extruded FFRB	FFRB	Extruded DDGS	DDGS
DM	93.35	92.29	91.57	87.91
OM	85.00	83.75	85.85	82.47
GE, MJ/kg	20.46	19.94	20.47	19.85
CP	14.70	14.09	28.70	27.25
EE	17.33	17.14	11.12	11.16
NDF	19.61	21.02	28.53	30.98
ADF	7.71	8.68	8.73	9.54
Ash	8.35	8.54	5.73	5.43
Indispensable AA				
Arg	1.01	0.98	0.99	1.04
His	0.35	0.35	0.73	0.67
Ile	0.56	0.45	1.09	0.96
Leu	0.97	0.88	3.30	3.00
Lys	0.67	0.68	0.86	0.79
Met	0.28	0.28	0.56	0.55
Phe	0.63	0.59	1.33	1.25
Thr	0.50	0.50	1.00	0.92
Trp	0.16	0.18	0.17	0.17
Val	0.79	0.77	1.42	1.26
Dispensable AA				
Ala	0.86	0.84	2.08	1.89
Asp	1.15	1.15	1.69	1.58
Cys	0.27	0.27	0.48	0.47
Glu	1.79	1.67	4.90	4.34
Gly	0.74	0.72	1.06	0.98
Pro	0.38	0.50	1.92	1.76
Ser	0.59	0.57	1.24	1.16
Tyr	0.46	0.43	0.92	0.83

^1^ AA, amino acid; DM, dry matter; OM, organic matter; GE, gross energy; CP, crude protein; EE, ether extract; NDF, neutral detergent fiber; ADF, acid detergent fiber; FFRB, full-fat rice bran; DDGS, distiller dried grains with solubles. ^2^ Data are the mean of duplicate analyses of each ingredient.

**Table 2:** Ingredient compositions of the experimental diets (Exp. 1) (%, as-fed basis) ^1^.

Item %	Basal Diet	Extruded FFRB	FFRB	Extruded DDGS	DDGS
Ingredient composition, as-fed basis					
Corn	96.90	67.83	67.83	67.83	67.83
FFRB	-	29.07	29.07	-	-
DDGS	-	-	-	29.07	29.07
Dicalcium phosphate	1.70	1.70	1.70	1.70	1.70
limestone	0.60	0.60	0.60	0.60	0.60
Salt	0.30	0.30	0.30	0.30	0.30
Vitamin–mineral premix ^2^	0.50	0.50	0.50	0.50	0.50
Total	100.00	100.00	100.00	100.00	100.00
Analyzed composition					
DM	89.41	90.46	89.38	90.44	90.47
OM	85.94	84.67	83.89	86.27	85.80
GE, MJ/kg	16.28	17.16	17.11	17.46	17.05
CP	8.55	10.28	9.94	14.35	14.10
EE	1.00	3.93	3.42	2.30	2.50
NDF	9.39	11.93	12.20	15.28	15.87
ADF	2.38	3.79	4.23	4.11	4.40
Ash	3.46	5.79	5.50	4.97	4.65

^1^ FFRB, full-fat rice bran; DDGS, distillers dried grain with solubles; DM, dry matter; OM, organic matter; GE, gross energy; CP, crude protein; EE, ether extract; NDF, neutral detergent fiber; ADF, acid detergent fiber. ^2^ Premix provided the following per kilogram of feed: vitamin A, 12,000 IU; vitamin D3, 2500 IU; vitamin E, 30 IU; vitamin K3, 3.0 mg; vitamin B1, 2.5 mg; vitamin B2, 4.0 mg; vitamin B6, 3.0 mg; vitamin B12, 12.0 μg; nicotinic acid, 40.0 mg; thiamine, 3.0 mg; Riboflavin, 6.0 mg; D-pantothenic acid, 15.0 mg; folic acid, 1.2 mg; biotin, 50.0 μg; Fe, 90.0 mg; Cu, 75.0 mg; Zn, 75.0 mg; Mn, 40.0 mg; I, 0.4 mg; Se, 0.3 mg.

**Table 3:** Ingredient compositions and nutrient levels of the experimental diets (Exp. 2) ^1^

Item %	N-Free	Extruded FFRB	FFRB	Extruded DDGS	DDGS
Corn starch	68.90	34.40	34.40	34.40	34.40
WRB	-	40.00	40.00	-	-
DDGS	-	-	-	40.00	40.00
Cellulose acetate	4.00	-	-	-	-
Soybean oil	3.00	3.00	3.00	3.00	3.00
Dicalcium phosphate	1.60	1.00	1.00	1.00	1.00
Magnesium oxide	0.10	-	-	-	-
limestone	1.00	0.50	0.50	0.50	0.50
Salt	0.30	0.30	0.30	0.30	0.30
Vitamin–mineral premix ^2^	0.50	0.50	0.50	0.50	0.50
Chromic oxide	0.30	0.30	0.30	0.30	0.30
Total	100.00	100.00	100.00	100.00	100.00
Analyzed composition					
CP	0.96	6.25	5.76	11.25	11.00
Indispensable AA					
Arg	0.03	0.50	0.49	0.39	0.40
His	0.03	0.24	0.14	0.29	0.29
FIle	0.02	0.34	0.22	0.43	0.42
Leu	0.06	0.49	0.39	1.34	1.24
Lys	0.07	0.39	0.30	0.29	0.32
Met	0.01	0.12	0.11	0.22	0.21
Phe	0.06	0.45	0.26	0.55	0.52
Thr	0.04	0.32	0.21	0.42	0.39
Trp	0.01	0.06	0.06	0.07	0.07
Val	0.06	0.42	0.38	0.58	0.54
Dispensable AA					
Ala	0.05	0.59	0.38	0.84	0.79
Asp	0.03	0.57	0.47	0.74	0.65
Cys	0.01	0.11	0.10	0.18	0.18
Glu	0.06	0.84	0.73	1.97	1.85
Gly	0.03	0.67	0.31	0.45	0.42
Pro	0.05	0.54	0.25	0.85	0.89
Ser	0.04	0.57	0.25	0.51	0.49
Tyr	0.04	0.38	0.17	0.38	0.38

^1^ FFRB, full-fat rice bran; DDGS, corn distillers dried grain with solubles; CP, crude protein. ^2^ Premix provided the following per kilogram of feed: vitamin A, 12,000 IU; vitamin D_3_, 2500 IU; vitamin E, 30 IU; vitamin K_3_, 3.0 mg; vitamin B_1_, 2.5 mg; vitamin B_2_, 4.0 mg; vitamin B6, 3.0 mg; vitamin B_12_, 12.0 μg; nicotinic acid, 40.0 mg; thiamine, 3.0 mg; Riboflavin, 6.0 mg; D-pantothenic acid, 15.0 mg; folic acid, 1.2 mg; biotin, 50.0 μg; Fe, 90.0 mg; Cu, 75.0 mg; Zn, 75.0 mg; Mn, 40.0 mg; I, 0.4 mg; Se, 0.3 mg.

**Table 4:** Effects of available energy and digestibility of extruded or non-extruded FFRB and DDGS ^1^.

Items	Main Effect				SEM	*p*-Values		
	FFRB	DDGS	Extruded	Non-Extruded		Processing	Ingredient Type	Interaction
DE	14.55	15.14	15.86	13.84	0.38	<0.01	0.39	0.35
ME	14.21	14.39	15.24	13.36	0.37	<0.01	0.37	0.91
ME/DE	0.97	0.94	0.96	0.95	0.01	0.52	0.07	0.17
ATTD, %								
GE	74.82	73.33	78.60	69.55	1.92	<0.01	0.57	0.14
DM	62.28	65.04	66.55	60.77	1.54	0.02	0.93	0.39
OM	66.61	66.12	69.31	63.41	1.51	0.01	0.23	0.35
CP	57.41	72.82	68.54	61.69	2.88	0.06	0.02	0.41
NDF	27.56	49.28	41.79	35.04	3.49	0.02	<0.01	0.24
ADF	21.75	48.25	37.77	32.24	3.85	0.07	<0.01	0.20

^1^ SEM, standard error of the mean; DE, digestible energy; ME, metabolizable energy; GE, gross energy; DM, dry matter; OM, organic matter; CP, crude protein; EE, ether extract; NDF, neutral detergent fiber; ADF, acid detergent fiber; FFRB, full-fat rice bran; DDGS, corn distillers dried grain with solubles. The corn basal diet was only used to calculate energy contents of tested ingredients; therefore, the raw data for a corn basal diet are not shown.

**Table 5:** Apparent ileal digestibility of crude protein and amino acids in extruded and non-extruded FFRB and DDGS fed to growing pigs ^1^ (%).

Items	Main Effect				SEM	*p*-Values		
	FFRB	DDGS	Extruded	Non-Extruded		Processing	Ingredient Type	Interaction
CP	60.16	57.91	60.79	57.28	2.98	0.42	0.34	0.36
Indispensable AA								
Arg	72.66	61.73	69.55	64.86	1.95	0.32	<0.01	0.53
His	68.84	71.75	75.49	65.09	1.62	<0.01	0.06	<0.01
Ile	65.01	75.43	71.64	68.81	1.47	0.33	<0.01	0.46
Leu	71.31	83.95	84.45	70.81	2.22	<0.01	<0.01	<0.01
Lys	66.64	48.67	62.08	53.24	3.04	0.01	<0.01	<0.01
Met	70.94	82.16	79.59	73.51	1.52	<0.01	<0.01	<0.01
Phe	72.60	78.85	83.95	67.50	2.36	<0.01	<0.01	<0.01
Thr	55.27	62.15	64.65	52.78	2.20	<0.01	0.01	<0.01
Trp	55.72	49.74	53.19	52.28	2.52	0.58	0.65	0.17
Val	74.77	71.86	79.32	67.31	1.77	<0.01	0.06	<0.01
Dispensable AA								
Ala	66.63	74.78	77.73	63.67	2.13	<0.01	<0.01	<0.01
Asp	66.48	63.70	73.80	56.39	2.56	<0.01	0.44	<0.01
Cys	48.70	60.50	56.89	52.33	2.15	0.35	<0.01	<0.01
Glu	74.22	80.82	83.59	71.45	1.81	<0.01	<0.01	<0.01
Gly	49.95	34.67	46.21	38.41	3.46	0.01	0.04	0.20
Ser	63.71	69.05	74.85	57.91	2.57	<0.01	0.01	<0.01
Tyr	71.63	79.69	82.29	69.03	2.11	<0.01	<0.01	<0.01

^1^ SEM, standard error of the mean; FFRB, full-fat rice bran; DDGS, corn distillers dried grain with solubles; CP, crude protein.

**Table 6:** Standardized ileal digestibility of crude protein and amino acids in extruded and non-extruded FFRB and DDGS fed to growing pigs ^1^ (%).

Items	Main Effect				SEM	*p*-Values		
	FFRB	DDGS	Extruded	Non-Extruded		Processing	Ingredient Type	Interaction
CP	70.22	75.86	74.51	71.57	2.16	0.47	0.10	0.32
Arg	87.19	80.82	85.96	82.05	1.68	0.41	0.06	0.20
His	86.75	82.44	87.54	81.66	1.33	0.07	0.04	0.55
Ile	84.19	86.25	86.06	84.38	0.97	0.74	0.21	0.83
Leu	85.76	90.11	92.10	83.77	1.22	<0.01	<0.01	<0.01
Lys	82.12	68.25	77.86	72.51	2.47	0.2	<0.01	0.02
Met	77.64	86.05	84.57	79.12	1.24	<0.01	<0.01	0.07
Phe	84.26	87.49	90.96	80.79	1.54	<0.01	0.03	<0.01
Thr	84.88	82.26	84.87	82.27	1.54	0.75	0.42	0.71
Trp	79.23	71.45	74.98	75.70	2.88	0.43	0.36	0.78
Val	87.74	84.12	89.21	82.64	1.24	<0.01	0.01	<0.01
Ala	83.42	85.50	88.60	80.32	1.41	0.01	0.34	0.32
Asp	81.81	78.78	85.19	75.40	1.83	<0.01	0.2	0.01
Cys	71.72	73.84	74.03	71.53	1.66	0.88	0.38	0.18
Glu	88.05	88.28	91.59	84.73	1.14	<0.01	0.83	0.26
Gly	72.41	64.91	74.70	62.62	3.94	0.30	0.59	0.84
Ser	84.39	83.85	88.09	80.16	1.57	0.03	0.71	0.16
Tyr	85.47	88.63	90.80	83.30	1.26	<0.01	<0.01	<0.01

^1^ SEM, standard error of the mean; FFRB, full-fat rice bran; DDGS, corn distillers dried grain with solubles; CP, crude protein.

## Data Availability

The original contributions generated for this study are included in the article; further inquiries can be directed to the corresponding author.
